# Attitudes of Undergraduate Nursing Students towards Patient Safety: A Quasi-Experimental Study

**DOI:** 10.3390/ijerph18041429

**Published:** 2021-02-03

**Authors:** Nuria Cantero-López, Víctor M. González-Chordá, María Jesús Valero-Chillerón, Desirée Mena-Tudela, Laura Andreu-Pejó, Rafael Vila-Candel, Águeda Cervera-Gasch

**Affiliations:** 1 Department of Nursing, Faculty of Health Sciences, Universitat Jaume I, 12071 Castellón, Spain; al362075@uji.es (N.C.-L.); chillero@uji.es (M.J.V.-C.); dmena@uji.es (D.M.-T.); pejo@uji.es (L.A.-P.); cerveraa@uji.es (Á.C.-G.); 2 Department of Obstetrics and Gynaecology, La Ribera University Hospital, FISABIO, Crta. Corbera km 1, 46600 Valencia, Spain; vila_rafcan@gva.es; 3 Department of Nursing, Faculty of Nursing and Podiatry, University of Valencia, Jaume Roig, s/n, 46010 Valencia, Spain

**Keywords:** nurse education, patient safety, competence, attitude, critical incident technic, root cause analysis

## Abstract

Improving nursing students’ attitudes towards patient safety is a current and relevant topic. This study aims to evaluate the effectiveness of an educational intervention based on critical incident and root cause analysis (RCA) techniques regarding attitudes towards patient safety in nursing students. A quasi-experimental before and after study was developed between January 2018 and December 2019 in a sample of 100 nursing students at Universitat Jaume I (Spain). The intervention was developed in two phases. Phase I was at university, where students applied the RCA technique in a real case. Phase II took place during clinical practice. Students used critical incidents to identify a risk situation for the patients and applied RCA to detect its root causes. The measurement of attitudes was performed with the Attitudes to Patient Safety Questionnaire (APSQ-III). The global score of the questionnaire in the baseline measurement was 3.911 (±0.335), in the intermediate measurement it was 4.031 (±0.337) and in the final measurement it was 4.052 (±0.335), with significant differences (*p* = 0.03). However, intra-group differences were observed in the final measurement (*p* = 0.021). The teamwork dimension had the highest mean score on all three measures and the notification dimension had the lowest mean scores. An educational intervention combining critical incident and RCA techniques can improves nursing students’ attitudes toward patient safety.

## 1. Introduction

Since the beginning of the century, there has been growing concern with the education of future health professionals regarding the knowledge, skills and attitudes necessary to improve their competencies in patient safety. The World Health Organization (WHO) [1] and different agencies in the United States [2,3], Australia [4], Canada [5] and Europe [6] have developed competency profiles for patient safety that health science students must acquire, including nursing students.

However, around the world nursing degree curricula do not assign the necessary importance to competencies related to patient safety [7,8,9,10] ⁠and it is necessary to advance the design and evaluation of more effective educational interventions to improve knowledge, skills and attitudes regarding patient safety [7,11], both in university classrooms and clinical settings [12,13].

In the literature, different approaches can be found to improve the competencies in patient safety of nursing students. Some authors use traditional approaches with master classes [14] or structured courses [15]. Other authors introduce digital recordings in their courses [16,17] or use instructional methods such as the flipped classroom [18,19]. On the other hand, Lee and Quinn [13] conclude that clinical simulation, technological aids and online learning modules improve the safety competencies of nursing students. However, more research is needed to identify which educational strategies are the most appropriate, integrating theoretical and clinical learning [11,12].

During clinical practices, nursing students may witness situations that put patient safety at risk and may make errors that cause damage to their patients [20]. The most frequent errors are related to medication administration, the improper handling of equipment and biological accidents [21,22]. Despite the importance of knowing these errors to improve healthcare processes [23], nursing students are reluctant to report these situations for fear of the consequences [24], and it is necessary to improve their attitudes towards teamwork and open communication or error reporting [25]. In fact, Kong et al. [26] found an association between favorable attitudes of nursing students towards patient safety and the execution of related behaviors—for example, error notification—which also occurs when they are professionals [27].

Therefore, it is important to improve the attitudes of nursing students towards patient safety, although attitudes are probably one of the most complex components to introduce and evaluate in the teaching–learning process [28]. Student mistakes during practice can be considered learning opportunities; techniques initially developed in the field of engineering and industrial safety, such as critical incident [29] or root cause analysis (RCA) [30], can be used as teaching strategies to improve the attitudes of undergraduate nursing students towards patient safety.

In 1950, Flanagan defined a critical incident as “any observable human activity that is complete enough in itself to allow inferences and predictions about the person performing the act” and developed this retrospective technique of analysis of human behavior [31]. Since then, the critical incident technique has been used in nursing education to optimize learning from real experiences lived by students—for example, in the development of critical thinking, cultural and ethical aspects [29], or learning from evidence-based practice [32].

On the other hand, root cause analysis (RCA) is a reactive technique based on the assumption that problems are best solved by trying to correct or eliminate root causes, rather than simply trying to solve immediate consequences [33]. Several authors propose the use of RCA as a technique to teach patient safety to nursing students [3,30], and other authors [34] conclude that this technique promotes critical thinking and a positive attitude in students when real cases are analyzed.

These techniques show favorable results when they are used as teaching methods, but a limited number of studies have evaluated the relationship between their effect on attitudes and patient safety in nursing students. Thus, the main objective of this study was to evaluate the effectiveness of an educational intervention based on critical incident and RCA techniques regarding attitudes related to patient safety in undergraduate nursing students at the Universitat Jaume I (Spain).

## 2. Materials and Methods

### 2.1. Design and Settings

A quasi-experimental before and after study was carried out in third-year students of the degree in nursing at Universitat Jaume I (Spain). The study was conducted between January 2018 and December 2019.

### 2.2. Population and Sample

The nursing degree at Universitat Jaume I has 360 students. The study population was made up of third-year students enrolled in the subject “Management of care in the socio-sanitary field” in the academic years 2017–2018 and 2018–2019 (*N* = 108). This subject has a total of six credits within the European Credit Transfer System (ECTS). The theoretical contents deal with aspects related to management models, leadership, quality of care and patient safety. The period of clinical practice is carried out in adult hospitalization units, where students carry out an activity that allows applying the theoretical contents of the subject in the healthcare reality.

Through a convenience sampling, all the students enrolled in this subject were included. Those who did not complete the clinical practice period and those who did not agree to participate in the study were excluded.

### 2.3. Educational Intervention

**Figure 1:**
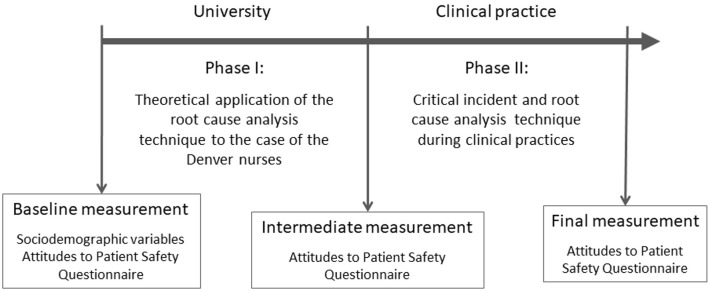
Study sequence.

The educational intervention was based on RCA and critical incident techniques and was carried out in two phases, developing in the same way in the two academic years considered (Figure 1). Phase I was carried out in the classroom by the teacher responsible for the subject and a member of the research team. The duration was 120 min. With no prior structured knowledge about patient safety, groups of five students applied the RCA technique in a real case related to errors in the administration of medication known as “The case of the Denver nurses” [35]. This case is based on real events and deals with the chain of human errors and system failures that occurred from the admission of a pregnant woman to the death of the baby due to an error in the administration of medication. The exercise was based on teaching materials on patient safety prepared by the Spanish Ministry of Health, Social Services and Equality [36]. In addition, the exercise included a series of questions to delve into the basic principles related to patient safety (e.g., whose fault was it?). The case was resolved and a guided reflection was carried out on the basic principles of patient safety.

Phase II was carried out during clinical practice. Individually, each student used the critical incident technique to analyze a situation experienced during clinical practices that posed a risk to patient safety and applied the RCA technique to that situation. Examples of critical incidents related to patient safety during clinical practices include errors in the administration of medication, the fall of a patient during mobilization or a failure to rescue. Likewise, each student carried out a bibliographic search to propose measures that could reduce or eliminate the root causes of this situation. The students had a guide to prepare the work and the support of the teaching staff of the subject.

### 2.4. Data Collection Instrument

Attitudes towards patient safety were assessed with the Attitudes to Patient Safety Questionnaire (APSQ-III). The original version of the APSQ-III was developed by Carruthers et al. [37] for medical students and Lamponi et al. [38] validated it in Spanish. In this study, a version for nursing students adapted and validated in a previous study pending publication was used. This version is composed of 22 items measured with a 5-point Likert scale (1, strongly disagree; 5, strongly agree) distributed in six dimensions that explained 53.82% of the variance in the exploratory factor analysis (Responsibility; Organization and communication; Teamwork; Training; Notification; Awareness). A confirmatory factor analysis indicates an adequate fit between the model and the data (χ2 = 366; *p* < 0.001; χ2/df = 1.886; RMSEA = 0.07; 95% Cl = 0.059–0.081; CFI = 0.885). The questionnaire offered an adequate internal consistency (α = 0.808) and a good intra-observer reliability (*ICC* = 0.792). A higher score on the questionnaire means better attitudes towards patient safety.

In addition, sociodemographic variables were collected, such as age, sex (female; male), academic year (2017–2018; 2018–2019), previous studies related to health sciences (no; university; professional training; others) and employment status (unemployed; active in the health field; not active in the health field).

### 2.5. Data Collection Process

Data collection took place between February and July, in the 2017–2018 and 2018–2019 academic years. A baseline measurement was performed, prior to the intervention, where sociodemographic variables were also collected. Likewise, an intermediate measurement was made after Phase I of the intervention and a final measurement after Phase II, at the end of the clinical practices. In the baseline measurement, the students received information about the objectives and methodology of the study, as well as its voluntary and anonymous nature. In all three measurements, scheduled classes were used to collect data.

### 2.6. Data Analysis

A descriptive analysis of the variables was carried out according to their nature. The effectiveness of the intervention was determined with the Friedman test for paired data, comparing the global scores of the questionnaire and its dimensions in the three measurements (baseline, intermediate and final) and with the Wilcoxon test for paired data, comparing two by two at each measurement.

In addition, a bivariate analysis was performed to determine if there were significant differences in the global score of the questionnaire in the three measurements (baseline, intermediate and final) based on sociodemographic variables. After confirming that the application conditions of the parametric tests (homoscedasticity and normality) were not met, the non-parametric Mann–Whitney U test was used for two groups and the Kruskal–Wallis test for three or more groups. The qualitative variables were analyzed with the Chi-square test (X2). The standard deviation (*SD*) of the quantitative variables’ mean was calculated. Statistical analysis was performed with SPSS program version 23.0 (IBM SPSS Statistics for Windows, IBM Corp., Armonk, NY, USA). The significance level was set at *p* < 0.05.

### 2.7. Ethical Considerations

This study was approved and authorized by the Nursing Department Council of the Universitat Jaume I (project reference 19CO1/000156, 5 September 2019). In the baseline measurement, the participants were informed of the study objectives and its voluntary nature. Informed consent was obtained from all the subjects involved in the study. A dissociation code was used to relate the three measurements and ensure the anonymity of the participants. The study was designed and executed in accordance with Spanish Organic Law 03/2018 on Protection Personal Data and Guaranteeing Digital Rights and the ethical principles of the Declaration of Helsinki (beneficence, non-maleficence, autonomy and justice).

## 3. Results

### 3.1. Sample Characteristics

Eight participants were excluded because they did not adequately fill in the dissociation code in any of the three rounds. Thus, the final sample consisted of a total of 100 students. Fifty percent of the students belonged to the 2017–2018 academic year. The mean age of the sample was 22.54 ± 5.92 years and 78.8% (*n* = 79) were women. There were no significant differences when comparing the participants according to academic year (Table 1).

### 3.2. Effectiveness of the Educational Intervention

The global score of the questionnaire in the baseline measurement was 3.91 ± 0.34 points, increasing in the intermediate measurement (4.03 ± 0.34) and in the final measurement (4.05 ± 0.34). The Friedman test confirmed the overall effectiveness of the intervention when comparing the three measurements (*p* = 0.03). Likewise, statistically significant differences were found between the baseline and the intermediate measurement (*p* = 0.008), but not between the intermediate and the final measurement (*p* = 0.664).

Table 2 shows the analysis of the scores obtained in the different dimensions. Responsibility (*p* = 0.004), Training (*p* = 0.001) and Awareness (*p* = 0.047) showed statistically significant differences when comparing the mean scores of the three measurements. However, no dimension showed significant differences when comparing the mean score of the intermediate and the final measurement (*p* > 0.05). On the other hand, the Teamwork dimension obtained the highest score in the three measurements, while the lowest scores were for the Notification dimension.

### 3.3. Analysis of the Global Score of the Questionnaire According to Sociodemographic Variables

Table 3 shows the results of the questionnaire score according to the sociodemographic variables. On the one hand, no significant differences were observed as a function of sex in any of the three measurements (*p* > 0.05). On the other hand, the students who worked in the health field obtained a significantly lower score in the three measurements (*p* < 0.05) and this score decreased throughout the educational intervention, although not significantly (*p* = 0.223). In addition, students without previous studies related to health sciences obtained higher scores in the three measurements, although significant differences were only found in the intermediate measurement (*p* = 0.042). Finally, it was observed that the students of the 2018–2019 academic year obtained slightly higher scores in the three measurements, although there were only statistically significant differences in the final measurement (*p* = 0.021).

## 4. Discussion

The main findings of this study showed a significant increase in the global score of the APSQ-III questionnaire adapted for nursing students throughout the three measurements, so that it can be affirmed that this educational intervention based on the RCA and critical incident techniques significantly improved the attitudes towards patient safety. Other authors [18,19,39] also carried out educational interventions aimed at improving the patient safety competencies of nursing students, but it is difficult to compare our results, as other studies with a similar educational intervention were not found and most of them evaluate attitudes together with knowledge and skills and are not specific. Furthermore, there is variability in the instruments used to evaluate the effectiveness of educational interventions. For example, Breitkreuz et al. [39] compared the use of videos and simulation to improve nursing students’ attitudes towards patient safety, concluding that simulation was more effective. Kim et al. [19] compared the flipped classroom methodology with regular teaching in a sample of 75 nursing students. The authors concluded that their intervention managed to significantly improve knowledge and skills related to patient safety, but not attitudes, mainly in final year students. Maxwell et al. [18] obtained similar results using the flipped classroom methodology, although in their study there was no control group and different instruments were used to evaluate the effectiveness of the interventions.

Mansour et al. [14] evaluated whether knowledge and attitudes about patient safety improved in a sample of 141 English nursing students after two master classes and facilitated group work, but the results of this pre-post-intervention study were not significant. Another study with 59 Dutch nursing students obtained satisfactory results after applying a structured program on patient safety, but the authors used an ad hoc questionnaire to globally assess the competencies established by the WHO and not the acquisition of knowledge, skills and attitudes [15]. Other authors also did not find significant differences in attitudes towards patient safety when they evaluated different educational interventions [40,41].

In our study, Phase I of the educational intervention took place in the classroom, while Phase II was carried out during clinical practice. It should be mentioned that the score of the questionnaire increased slightly between the intermediate and the final measurement, although not significantly, indicating that the clinical practices did not negatively affect students’ attitudes. On the one hand, our students receive structured training on patient safety for the first time in the subject where the intervention was carried out, and this could explain why Phase I of the intervention significantly improved their attitudes towards patient safety. On the other hand, Lukewich et al. [42] observed that attitudes toward patient safety in nursing students decreased as their contact with clinical practices increased. It is possible that the level of safety culture of the units where students carry out the clinical practices [26], the quality of the learning environment [42] or the role played by clinical tutors as models for students [43] may influence these results. In the same way, these factors can help to explain the significant differences found in the final measurement when comparing the results between both courses, as practice units are awarded each year according to the centers’ addresses, and both the units and the clinical practice tutors may change. Future studies should consider the influence of these factors.

Despite this, integrating patient safety education into clinical practice and reducing the gap between theory and practice is a common recommendation to improve the patient safety competencies of nursing students. Although it is not clear what the best strategies for this are [9,12,13], the results of our study can help in this regard.

The version of the APSQ-III used in this study for nursing students has 22 items organized in six dimensions (Responsibility, Organization and Communication; Teamwork; Training; Notification; Awareness) that are directly related to patient safety. The dimensions Teamwork and Organization and Communication are related to a fundamental principle of patient safety, which is that errors in medical care depend more on failures in the system and in the design of processes than on human errors [44]. This is also related to the Responsibility and Awareness dimensions, although the aim here is to assess how students perceive responsibility for adverse effects and the need to communicate undesirable results to patients if they occur [45]. The Notification dimension refers to the attitude of students towards the reporting of errors to managers, which is a fundamental aspect in improving patient safety [46]. Finally, the Training dimension focuses on the importance of education in understanding the causes of errors and adverse effects [1,2,3,4,5,6]. Other questionnaires on patient safety in nursing students present similar structures and dimensions that address the same concepts [47].

The educational intervention evaluated in this study began with the analysis of a real case that affected patient safety. This case focuses on an error in the administration of medication, one of the main causes of adverse effects [48], and ends with the death of the patient. In the analysis of the case with the RCA technique, a chain of system failures and human errors were observed, mainly related to communication problems, one of the main root causes of errors [49]. The guided reflection after the analysis of the case made it possible to address aspects related to the organization of the processes and responsibility for errors, considering that initially the nurses were found guilty and, after the real analysis of the case with the RCA technique, it could be shown that they were not directly responsible for the death of the patient. In addition, during Phase II of the intervention, the students relied on real cases that they experienced during clinical practice to reflect on patient safety and apply the contents and techniques addressed in the classroom. Thus, the educational content used can influence nursing students’ attitudes towards patient safety and are linked to the dimensions of the APSQ-III.

In our study, the dimensions that obtained the highest scores were Organization and Communication and Teamwork, while the Notification and Responsibility dimensions obtained the lowest scores. These results partially coincide with those of other authors [50], where the best results were obtained in aspects related to communication and the worst in aspects related to teamwork. However, students carry out clinical practice with more experienced nurses and they are expected to be able to work in teams and communicate professionally with their colleagues and also with patients. In addition, students are expected to be cautious, ask the nurses when in doubt and notify them if they make a mistake. However, nursing students are unwilling to report adverse effects and their motives need to be understood in order to improve their attitudes towards reporting [24,25,26].

Regarding the influence of sociodemographic variables, several aspects should be discussed. On the one hand, only significant differences were observed in the APSQ-III score based on previous studies in the intermediate measurement. In addition, it was observed how the score decreased along the three measurements in students with previous professional training and how students with other types of training (for example, first aid courses or training as lifeguards) had the highest score. However, it must be taken into account that the majority of students did not have any type of previous training and that the other subsamples of this variable were not very representative, which could affect the results. Otherwise, the influence of previous studies of nursing students on their attitudes towards patient safety should be addressed in greater depth in future research, since no previous studies were found that considered this variable.

On the other hand, the variable employment situation had a significant influence on the three measures. Thus, students who were unemployed obtained higher scores than those who were working, possibly due to a greater availability of time to go to class and dedicate themselves to studying [41]. In addition, it is striking that the students who already worked in the health field obtained significantly lower scores than the rest and it is necessary to highlight that the score of these students who already worked in the health field decreased over the three measurements. These results deserve further study and are probably related to a low culture of patient safety in their work centers, although there are few studies on safety culture in our context [51]. These results have implications for future education if it is considered that, at least in our case, patient safety education is atomized into one subject and this may be insufficient for an adequate acquisition of competencies related to patient safety and, specifically, to develop or modify attitudes towards patient safety. Meanwhile, different authors recommend that competencies in patient safety be addressed throughout the different academic courses and are linked to experiences related to patient safety during clinical practice [9,43]. Finally, the results of this study should be viewed with caution due to their limitations. On the one hand, it is a quasi-experimental before and after study, with a non-randomized sample and no control group, which was carried out in a single institution, limiting the generalizability of the results. Furthermore, the long-term effectiveness of the intervention was not studied, and the results are based on self-report by the students, with a risk of information bias. Despite these limitations, the results of this study are of interest, as studies evaluating educational interventions to improve attitudes towards patient safety in nursing students are limited and few of them report a significant improvement in the attitudes of nursing students. In addition, the implications for practice should be considered, as improving nursing students’ attitudes towards patient safety should improve certain behaviors, such as reporting adverse effects, teamwork and communication with patients when they work as nurses. Thus, a direct impact on improving the quality of healthcare should be expected. The intervention offers an alternative with favorable results, although it is necessary to carry out studies with more robust experimental designs and larger samples to confirm the results obtained.

## 5. Conclusions

This quasi-experimental study evaluated the effectiveness of an educational intervention on attitudes towards patient safety in nursing students. The results showed a significant global improvement in the attitudes of the sample studied and attitudes remain positive when students are exposed to the reality of clinical practice. These results should be confirmed in future studies with more robust designs that consider factors such as the quality of the learning environment, the level of safety culture of the practice units or the influence of the practice tutors in the acquisition of skills related to patient safety.

## Figures and Tables

**Table 1:** Sociodemographic variables according to academic year (*N* = 100).

Variables		Academic Year		*p * ^1^
		2017–2018% (*n*)	2018–2019% (*n*)	
Employment situation (*n* = 92)	Not employed	34.8 (32)	37.0 (34)	0.224
	Not in the health area	10.9 (10)	7.6 (7)	
	In the health area	7.6 (7)	2.1 (2)	
Sex (*n* = 99)	Female	40.4 (40)	38.4 (38)	0.766
	Male	10.1 (10)	11.1 (11)	
Previous studies (*n* = 90)	No	36.7 (33)	37.8 (34)	0.182
	University	4.4 (4)	0 (0)	
	Professional training	11.1 (10)	8.9 (8)	
	Other	1.1 (1)	0 (0)	
**Variables**		**m (SD)**	**m (SD)**	***p *** **^2^**
Age (*n* = 100)		22.50 (4.31)	22.58 (7.23)	0.122

^1^ Chi-square test; ^2^ Mann–Whitney U test.

**Table 2:** Analysis of the dimensions of the questionnaire (*N* = 100).

Dimensions	BM ^1^	IM ^1^	FM ^1^	*p*		
	m (SD)	m (SD)	m (SD)	BM-IM-FM ^2^	BM-IM ^3^	IM-FM ^3^
Responsibility	3.42 (0.58)	3.77 (0.62)	3.81 (0.68)	0.004	<0.001	0.868
Organization y communication	4.32 (0.55)	4.38 (0.47)	4.41 (0.60)	0.341	0.517	0.830
Teamwork	4.45 (0.48)	4.44 (0.51)	4.42 (0.57)	0.820	0.915	0.576
Training	3.93 (0.49)	4.11 (0.50)	4.24 (0.53)	0.001	0.007	0.168
Notification	3.36 (0.92)	3.18 (0.98)	3.25 (0.82)	0.377	0.090	0.864
Awareness	4.02 (0.70)	4.28 (0.68)	4.18 (0.66)	0.047	0.001	0.055

^1^ BM: Baseline measurement; IM: Intermediate measurement; FM: Final measurement; ^2^ Friedman test; ^3^ Wilcoxon test.

**Table 3:** Analysis of the global score of the questionnaire according to the sociodemographic variables (*N* = 100).

Variables	Measurements (m; SD)					
	Baseline		Intermediate		Final	
	(m; SD)	*p*	(m; SD)	*p*	(m; SD)	*p*
Employment situation ^1^		0.016		0.011		0.044
Not employed	3.96 (0.32)		4.08 (0.33)		4.09 (0.40)	
Not in the health area	3.71 (0.37)		4.06 (0.35)		4.04 (0.47)	
In the health area	3.80 (0.24)		3.70 (0.19)		3.46 (0.54)	
Sex ^2^		0.524		0.527		0.639
Female	3.90 (0.32)		4.04 (0.34)		4.07 (0.37)	
Male	3.96 (0.39)		3.99 (0.35)		3.99 (0.57)	
Previous studies ^1^		0.490		0.042		0.229
No	3.93 (0.35)		4.10 (0.32)		4.11 (0.41)	
University	3.73 (0.15)		3.65 (0.14)		3.86 (0.0)	
Professional training	3.92 (0.33)		3.93 (0.35)		3.86 (0.54)	
Other	4.28 (0.0)		4.37 (0.0)		4.37 (0.0)	
Academic year ^2^		0.247		0.434		0.021
2017–2018 period	3.87 (0.38)		4.009 (0.347)		3.94 (0.48)	
2018–2019 period	3.95 (0.28)		4.06 (0.33)		4.16 (0.32)	

^1^ Kruskal–Wallis test; ^2^ Mann–Whitney U test.

## Data Availability

The data presented in this study are available on request from the corresponding author.
